# A cutting tool architecture designed to address the parasitic mechanisms consuming excess power during machining and manufacturing operations–A review-based study towards sustainable manufacturing

**DOI:** 10.1371/journal.pone.0300132

**Published:** 2024-04-16

**Authors:** Sweta Baruah, Balkrishna C. Rao

**Affiliations:** 1 Department of Engineering Design, Indian Institute of Technology, Chennai, Tamil Nadu, India; 2 Center for Materials Processing and Tribology, Purdue University, West Lafayette, Indiana, United States of America; indian institute of technology jammu, INDIA

## Abstract

Metal cutting has been extensively studied over the years for improving its efficacy, yet, parasitic mechanisms like chatter and tool wear continue to generate higher forces and energy consumption with poor surface integrity. To address these parasitic mechanisms, a single-point turning cutter design is proposed based on the physics-of-machining including chatter theory to achieve reduced power consumption during the cutting of various metallic alloys like Al-6061, Ti-6Al-4V and others used by critical sectors such as aerospace and automotive. The current work focuses on aspects of machining that effectively reduce parasitic forces feeding into cutting power. The proposed cutter amalgamates features such as optimum side and end cutting edge angles, smaller nose radius and textured rake face into the cutter-body. Such a design is further proposed for use with a mechanochemical effect on a recently discovered plastic flow mode called sinuous flow, which has been reported to bring down cutting forces significantly. Experimental and analytical tests on the cutter design features validate reduction of cutting forces and through that alleviate the tendency to chatter as well as bring about energy savings for cutting of Al 6061. The potential for reduced real-time power consumption makes this design-framework significant for multipoint milling cutters too. It will greatly facilitate frugal manufacturing to account for sustainability in manufacturing operations.

## Introduction

The mechanics of metal cutting have been extensively studied since the crucial efforts by Merchant and Shaw from plasticity theories [1, 2]. Machining operations, as popular workhorses of manufacturing, consume significant amounts of energy. These operations account for 15% of the value of all mechanical components manufactured globally [3]. Being a vital part of an industrial setup, they also have a notable contribution towards *green-house gas* (GHG) emissions and raise the atmospheric carbon dioxide levels. According to the annual energy review of the US *Energy Information Administration* (EIA), the industrial sector accounted for about 30% of the total energy consumption which was projected to exceed pre-pandemic levels by the end of 2022 [4]. Of this, manufacturing’s share was 90% and within manufacturing, machine tools consumed 75% of the power [5]. Growing concerns over global warming with the advent of climate change make it imperative for machining operations to be energy-efficient. Any effort to make cutting efficient should start with the cutter, which is significant to the ensuing deformation process.

**Fig 1:**
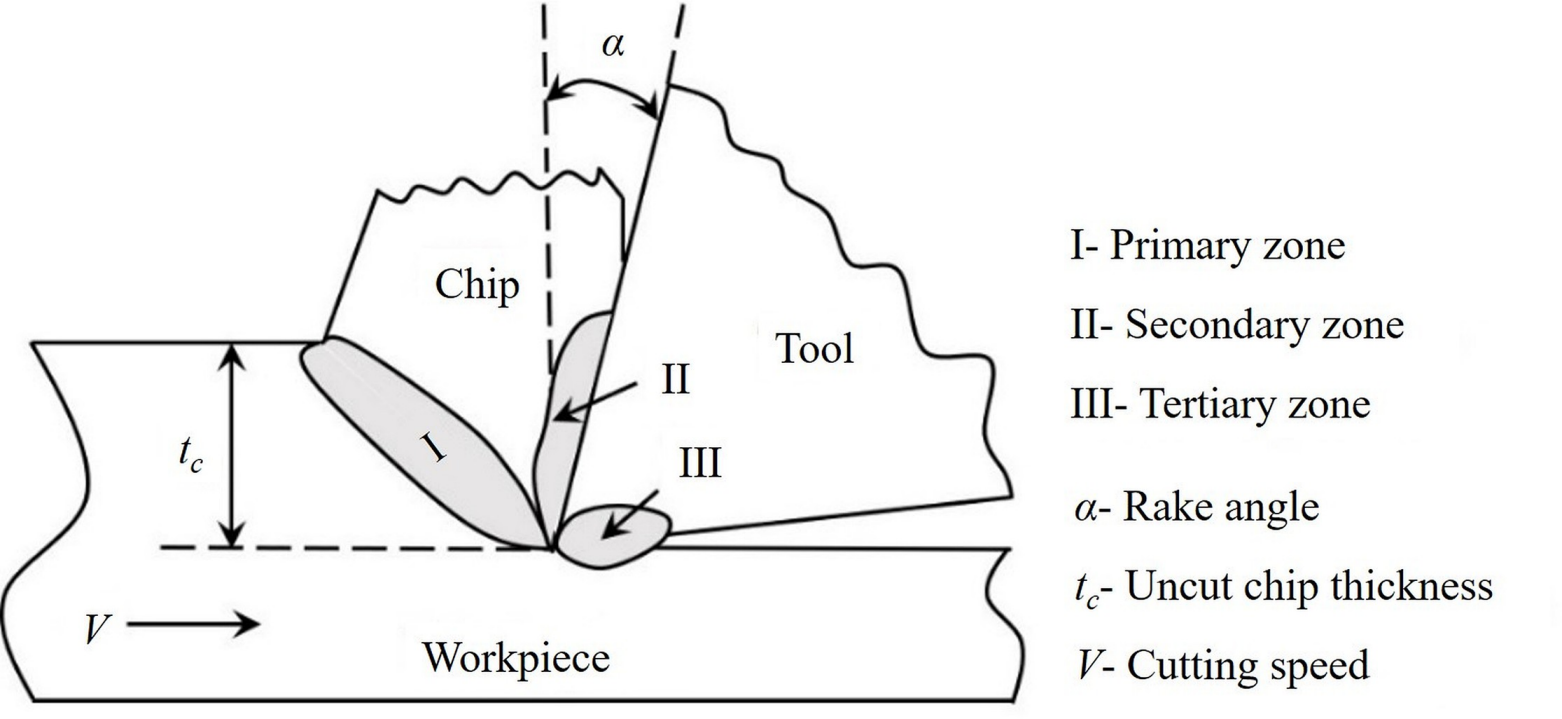
Schematic of a two-dimensional plane strain orthogonal machining process depicting the primary, secondary and tertiary deformation zones.

Fig 1 illustrates a two-dimensional schematic of a plane strain orthogonal machining process. Regions I, II and III represent primary, secondary and tertiary deformation zones, respectively. The workpiece enters with an *undeformed chip thickness* (*t*_*c*_) and gets sheared in the primary and secondary deformation zones dissipating energy as it forms a new machined surface. Some amount of energy also gets dissipated in the tertiary zone due to wear between the tool flank and the workpiece surface. Energy expended in actual cutting of the work material and non-cutting energy from sources such as and, not limited to, tertiary zone needs to be minimized by focussing on the mechanics of the metal cutting process involved therein.

The non-cutting energy expended in the tertiary zone mentioned above is an example of a parasitic mechanism. Parasitic forces are those forces generated during metal cutting that do not contribute to the actual chip formation process. These mechanisms may result from loss of cutting edge sharpness or tool wear; chatter; ploughing of an indenter-like tool through work-material; and flank face attrition aggravated by unfavourable surface integrity of the machined surface. Parasitic mechanisms consume power in excess of that required for cutting and hinder an otherwise efficient metal cutting process.

Chatter is a self-excited and self-sustaining vibratory phenomenon whose regenerative type is predominant in most metal cutting operations [6]. Regenerative chatter is the result of the interaction between the metal cutting process and the machine-tool structure dynamics. Chatter is thus, influenced by cutting tool geometry, tool-work material combination, cutting conditions and machine-tool-workpiece system dynamics. The ability to self-propagate makes chatter unbounded with time, thereby proving potentially catastrophic for the machine-tool structure, part and operator. Such unbounded vibrational motion is consequently accompanied by higher cutting forces leading to higher energy consumption. For controlling a parasitic phenomenon such as chatter, empirical testing is necessary for capturing tool-workpiece dynamics and also validating chatter predictions made using predictive modelling techniques. Tobias and Fishwick [7] examined the effect of chatter vibration on cutting tool life for turning and milling operations. They observed a significant reduction in tool life and increase in cutting forces due to chatter with the tool deteriorating under increasing vibration amplitude. Merritt [8] put forth a chatter theory capable of calculating stability borderlines for a machine-tool structure with ’*n*’ degrees of freedom, assuming no dynamic forces involved in the cutting process. Owing to complications that arose in using stability charts, Merritt simplified the stability criterion by relating it to the structure’s minimum dynamic stiffness. Siddhpura and Paurobally [9] presented different stability prediction techniques through theory and experiments. The resulting *stability lobe diagram* (SLD) from these and other endeavours [10, 11] has been widely used thereafter. These extensive studies concerning chatter that have been performed in the past involve either control theory together with experimental investigations to determine chatter-free machining conditions [8, 9] or the ancillary use of an active or passive damping device to absorb vibrations [12–14].

Tool wear is another parasitic mechanism that can occur either on the flank or rake-face region. The flank type occurs when the flank region rubs against the newly created machined surface while crater wear occurs on the rake face of the cutter due to tool and chip interaction. As a result, the amount of non-cutting energy expended due to these interactions and the ensuing cutting forces can be significant due to higher tool wear. Tool replacement or applying suitable coatings onto the tool surface are some prevalent measures adopted to incur less damage from flank or crater wear. Some commonly used tool coatings like *titanium carbide* (TiC), *titanium nitride* (TiN) and *alumina* (Al_2_O_3_) possess superior properties that can extend tool life even at high cutting speeds. Tool wear is intensified at elevated temperatures associated with higher cutting speeds. This intensification could be controlled using coolants or lubricants in the cutting zone to alleviate the cutting temperatures. However, coolants are discouraged from use, as they have severe environmental ramifications and inflict health hazards upon the operators.

High cutting forces, tool wear, cutting temperature and friction between contacting surfaces, all of which are parasitic in nature, have been controlled through changes in tool geometry. Shi and Ramalingam [15] studied the role of tool geometry in chip formation and associated chip flow for grooved tools. Grooves present on the tool face resulted in a smaller tool chip contact length reducing friction and cutting temperature. Jianxin et al. [16] performed dry cutting of hardened steel using self-lubricating cemented carbide tools with micro-holes on the rake and flank faces. These holes were filled with *molybdenum disulfide* (MoS_2_) solid lubricant, which formed a thin film between the tool face and workpiece during cutting. The formation of the lubricant film resulted in lesser cutting forces, lower tool wear and reduced friction compared to a conventional cutting tool. Xie et al. [17] performed dry cutting of a *titanium* (Ti-6Al-4V) alloy using a non-coated micro-grooved tool. They obtained the variation of cutting forces and also cutting temperature with micro-groove depth. The cutting forces and temperatures were seen to significantly decrease for the micro-grooved tool at an optimum micro-groove depth of 25 μm. This depth was large enough to accommodate an air gap between the tool rake and chip back faces. At the same time, it was smaller than the chip width to ensure chips did not accumulate on the grooves and slid over the rake face for removal from the cutting zone. Sharma and Pandey reviewed several research articles on sustainable manufacturing through the use of textured cutting inserts [18]. These inserts are designed to yield lower cutting forces, lower cutting temperatures, reduced tool wear and superior performance due to a commensurate reduction in tool-chip contact length combined with enhanced lubrication. Thus, tool geometry modifications can also improve the machinability of difficult-to-cut materials.

The cutting of ductile metals is known to generate continuous chips having thickness considerably greater than the undeformed chip thickness. Merchant [1] ascribed this large-scale transformation to occur by simple shear deformation and modelled the relevant features based on the assumption of steady-state one-dimensional laminar flow within the metal plasticity framework, see Fig 1. This laminar flow mode is homogenous and is characterized by a uniform and lamellar chip microstructure. However, plastic flow during metal cutting is not restricted to only homogenous laminar mode. Flow localization by shear banding is an example of a non-homogenous and highly non-uniform flow mode characterized by saw-tooth chip formation while cutting metallic alloys of Ti, Mg, Ni and others [19–21]. The occurrence and also stability of these flow modes are of primary importance in large-strain unconstrained deformation, especially near free surfaces. The factors influencing a particular flow mode comprise the work material’s initial deformation state, cutting tool geometry and the ambient environment of the work surface. The recent discovery of an unsteady plastic deformation mode called sinuous flow paves the way towards understanding the non-laminar and unstable flow mode and its underlying cause of trigger [20]. Suppression of this unsteady flow mode led to a large reduction of cutting forces brought about by a *mechanochemical* (MC) effect [22–24].

Several research groups have attempted various approaches to sustainable machining by focusing on energy efficiency at different levels of machine-tool systems [4]. However, there are limited energy efficiency studies on a machine-tool cutter as the primary device for minimizing energy consumption while achieving improved surface quality, form accuracy, increased throughput and longer tool life. This paper accordingly presents the design framework for energy-efficient cutters based on the principles of metal cutting. The paper aims to explore the cutting zone, where the shearing of work material to form a new surface expends high energy, to propose a cutter design that will intrinsically combat parasitic mechanisms to achieve energy efficiency. Methodologies, such as chatter control, geometric modifications, surface texture and MC effect, adopted for achieving this energy efficiency are described in detail for a single-point turning cutter with results from the literature to corroborate it’s working. There is a lacuna in literature in terms of individual geometric features of cutting edge angles and their effect on metal cutting efficiency or parasitic mechanisms. There are also limited studies dedicated to the cutter nose radius effects on the parasitic mechanisms discussed. In fact, the MC effect itself is a new and innovative concept and there have been no reports of it for turning or any other machining process. The current paper elaborates the design framework and presents a literature-based analysis of its performance in combating the various parasitic mechanisms. Such a cutter design will facilitate *frugal manufacturing* of metallic materials wherein the focus is on improving surface quality while consuming lesser energy affordably [25].

## Proposed cutter design

This section elaborates the features to be incorporated into our proposed cutter design for streamlining turning operations.

### Tool geometry

**Fig 2:**
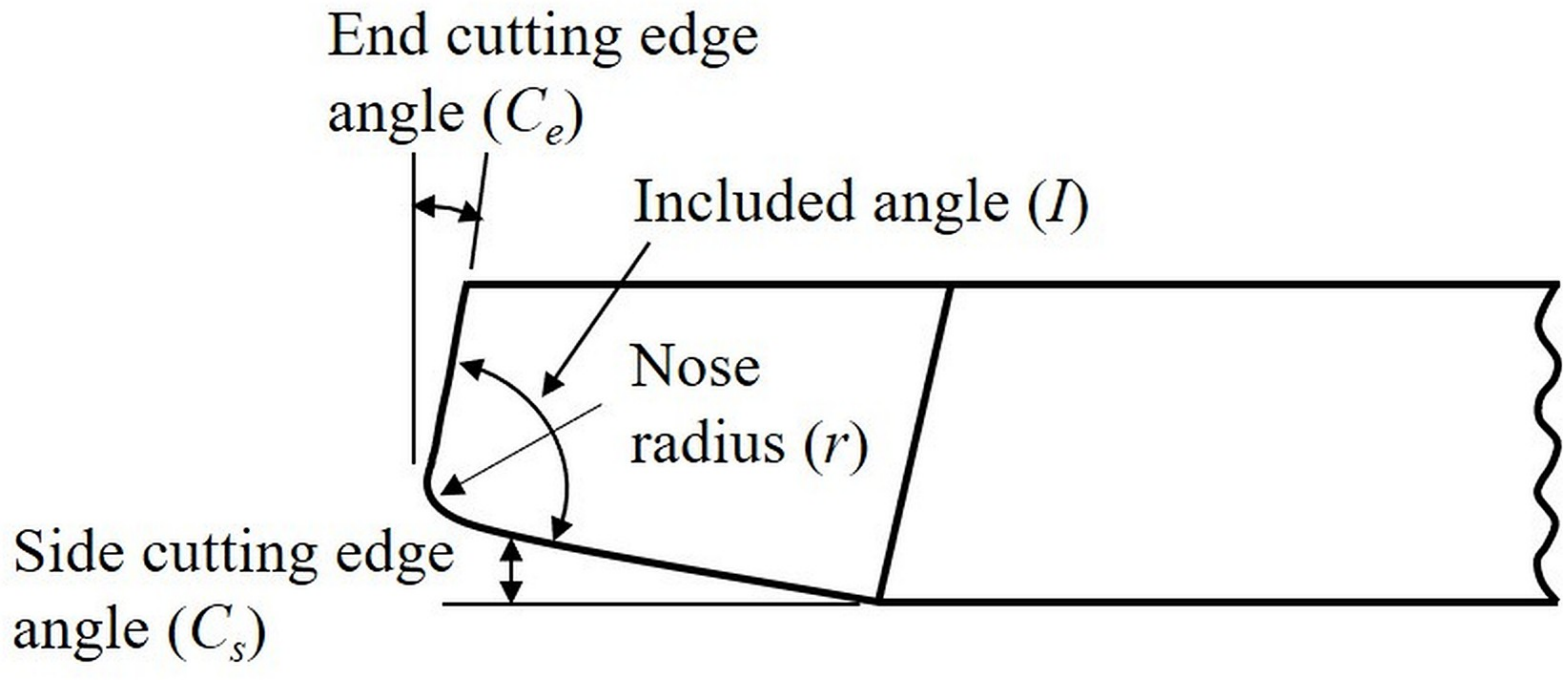
Top view of a single point turning cutter with associated tool geometry features.

Tool geometry features comprising *side* and *end cutting edge angles* and the nose radius of the cutter, shown in Fig 2, were selected for suitable modifications. This is because, the *side* and *end cutting edge angles*, sometimes collectively referred to as the *cutting edge angle*, affect chip formation, tool strength and cutting forces to various degrees [26]. This collective *cutting edge angle* is typically selected at around 15⁰ for several commonly used work materials [26]. In the current work, however, both these angles have been treated as separate entities and based on their functions in metal cutting, specific values are ascribed to them.

Much work has been done on features like rake angle (*α*), clearance angle (*γ*), etc to facilitate machinists and researchers to determine their values as per the work material and machining objectives [26–31]. On the other hand, there is very little knowledge or experimental data available that drafts the effect of the cutting edge angles, including side and end cutting edge angles, on metal cutting processes. The tool design is intended to envelop this lacuna and address as well as propose measures to counter parasitic forces. The design features proposed in the cutter will be incorporated with favorable *α* and *γ* values while selecting suitable cutting conditions as per requirement for cutting of soft and ductile metals like Al6061 and also harder metals like stainless steel, Ti6Al4V, etc. The proposed features have been selected keeping in mind the difference in metal cutting principles for different workpiece materials. At the same time, overlay between the parasitic mechanisms is also considered.

### Side cutting edge angle

**Fig 3:**
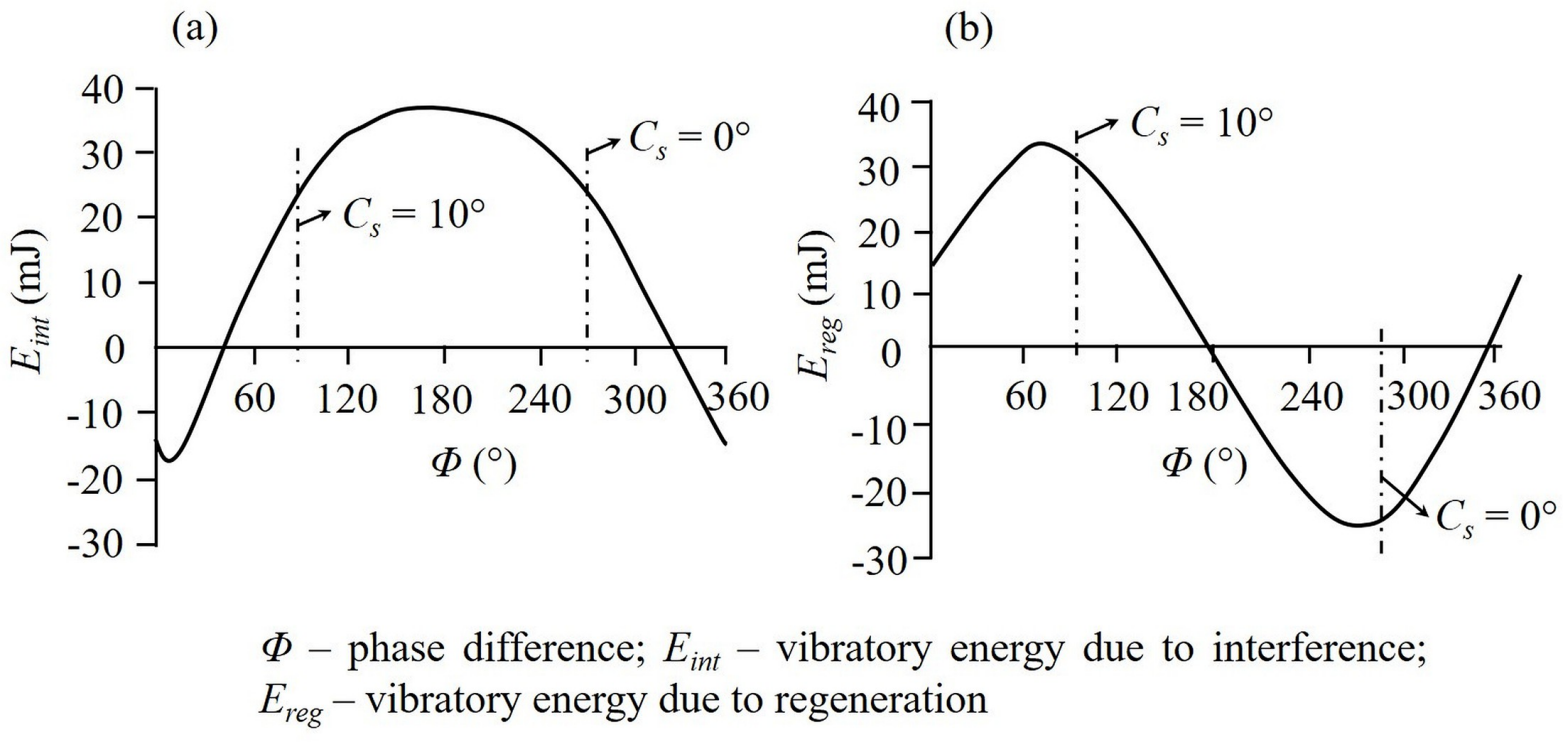
Vibratory energy due to (a) interference effect (*E*_*int*_) and (b) regenerative effect of undulations (*E*_*reg*_) [32].

The occurrence of chatter during machining is influenced by two factors. One, the interference between tool flank and workpiece and, second, the regenerative effect or waviness from the preceding cut affecting subsequent and current machined surfaces. The *side cutting edge angle* (*C*_*s*_) influences vibratory energy supply during the cutting process and through that impacts the regenerative effect and thereby, chatter vibration. *C*_*s*_, shown in Fig 2, facilitates entry of cutting tool into the workpiece and reduces any sudden impact experienced by the tool as it comes in contact with the workpiece [27]. Experimental and numerical investigations on fully developed chatter have facilitated a qualitative estimation of the vibratory energy supply due to interference and regenerative effects [32]. For a tool with zero *C*_*s*_, the flank face vigorously contacted the workpiece during the first half of tool motion as there was considerable negative relief between the contacting surfaces. The friction generated on the flank face caused cutting edge vibration, thereby supplying vibratory energy to the system during this period by interference between the tool flank and workpiece. Even though there was positive or slightly negative relief between the tool and work surfaces during the second half of tool motion, it had negligible influence on the vibratory energy supply. As a result, the net vibratory energy supplied by the interference effect for a tool with zero *C*_*s*_ was positive. Similar results were also obtained by Marui et al. [32] for a tool with *C*_*s*_ = 10⁰. Fig 3(A) shows the *vibratory energy supplied by the interference effect* (*E*_*int*_) as a function of *phase difference* (*Φ*) between two consecutive undulations on the work surface for these two cases.

The influence of regenerative effect on vibratory energy was studied by observations of the variation in dynamic cutting forces with the phase difference of cutting edge displacement of a tool cutting an undulated surface during chatter [32]. The magnitude of vibratory energy varied considerably with the phase difference between two consecutive undulations on the workpiece surface. However, experimental investigations for cutting revealed that vibratory energy was not supplied by the regenerative effect for a tool with zero *C*_*s*,_ as seen from the negative *vibratory energy from regenerative effect* (*E*_*reg*_) value in Fig 3(B). On the other hand, vibratory energy for a tool with *C*_*s*_ = 10⁰ was supplied by the regenerative effect, seen from the positive *E*_*reg*_ value in Fig 3(B), in addition to interference discussed above. These results corroborate the fact that an increase in *C*_*s*_ decreases the stability of the cutting process, which has also been observed by others [27, 32]. Marui and co-authors have reported that the critical cutting width at which the cutting process became unstable was smaller for the tool with *C*_*s*_ = 10⁰ when compared to the one with *C*_*s*_ = 0⁰. For a tool with *C*_*s*_ = 30⁰, the vibratory energy was supplied by the regenerative effect alone, without any influence from interference. Additionally, an increased *C*_*s*_ led to less sharp and pointed chatter marks on the workpiece surface when compared to those from *C*_*s*_ = 0⁰ [32].

The above results imply that *C*_*s*_ significantly impact chatter initiation and its subsequent propagation. For a sharp insert with a smaller nose radius, to be discussed subsequently, the contribution from the interference effect would be more prominent due to the engagement of the straight part of the cutting edge during cutting. Analysis of the above trend dictates that cutting with a tool having *C*_*s*_ = 20⁰ will nullify the interference effect between the tool flank and workpiece. At the same time, the influence of the regenerative effect will not be as significant as it is for *C*_*s*_ = 30⁰. Chatter can, thus, be largely minimized in the complete absence of the interference effect and moderate propagation due to the regenerative effect. In other words, although chatter cannot be completely eliminated, the tool having a *C*_*s*_ of 20⁰ would plausibly slow its onset. This *C*_*s*_ value along with a suitable *end cutting edge angle* (*C*_*e*_), elaborated in the next section, will collectively remain invariant to other tool geometry changes and as such, its chatter control aspect is essentially a fundamental effect.

### End cutting edge angle

**Fig 4:**
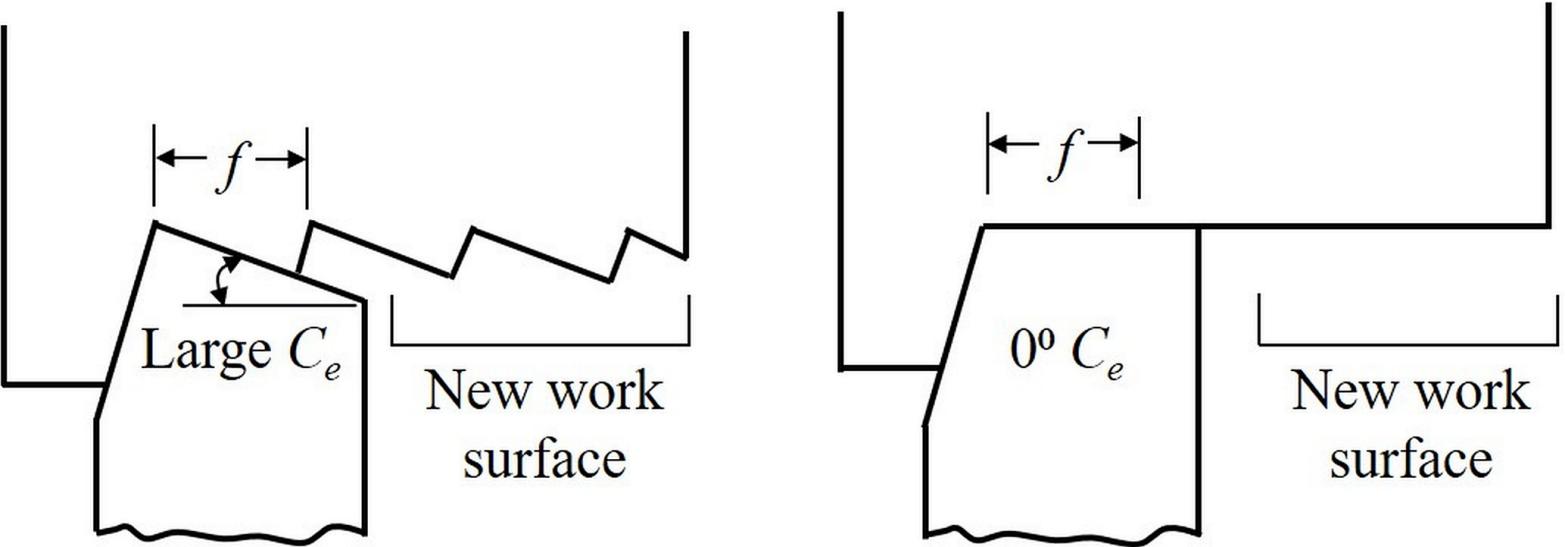
Effect of *end cutting edge angle* (*C*_*e*_) on the theoretical finish of machined surface for a single-point cutting tool [27].

The *end cutting edge angle (C*_*e*_*)*, another element of a single-point cutting tool geometry as shown in Fig 2, provides a clearance between the trailing edge of the tool and the freshly generated machined surface, thereby alleviating rubbing action and hence, friction between them [27]. It significantly impacts the quality of the machined surface, especially when a smaller nose radius is used which engages the end cutting edge in the creation of a new surface. Theoretically, a zero *C*_*e*_ should generate a perfectly smooth surface, see Fig 4. However, such a perfect surface finish is impeded in practice due to flaws in the tool, workpiece material and machining errors. Although the smallest possible value of *C*_*e*_ would impart a smooth finish, it would also strengthen back forces, thus leading to increased chatter that is unwanted. A *C*_*e*_ of 5⁰ is commonly recommended for commercial cutters to obtain a good surface quality in a wide range of materials [26]. But for sharp inserts, such a small *C*_*e*_ might lead to rubbing of the end cutting edge of the tool against the workpiece. In addition to initiating chatter-induced vibration, this involvement of the cutting edge with the workpiece surface could result in more heat generation that would reduce tool life.

On the other hand, increasing *C*_*e*_ decreases the *included angle* (I) between the side and end cutting edges, as shown in Fig 2. This decrease could again weaken the tooltip, with the effect being more pronounced for sharp cutting inserts. A higher *C*_*e*_ also results in higher surface roughness and will generate a finish not adequate to desired requirements. For a certain class of carbide inserts, a *C*_*e*_ of 15⁰ is generally recommended and applied [26]. This particular value again can be critical for an insert with a smaller nose radius in terms of surface quality requirements. The plausible loss in surface quality due to employment of a smaller nose radius cutter, to be discussed subsequently, can be compensated for by using an appropriate *C*_*e*_ value.

A *C*_*e*_ of 10⁰ is proposed in our cutter design. For a sharp insert, this angle will ensure that the cutting edge does not contact the workpiece surface during machining, thereby eliminating the above-discussed issues associated with a smaller *C*_*e*_. It is expected to reduce back forces and thus, minimize the tendency to chatter and also reduce heat generation and enhance tool life. Moreover, a *C*_*e*_ of 10° is not too high to adversely affect the quality of the machined surface.

### Nose radius

**Fig 5:**
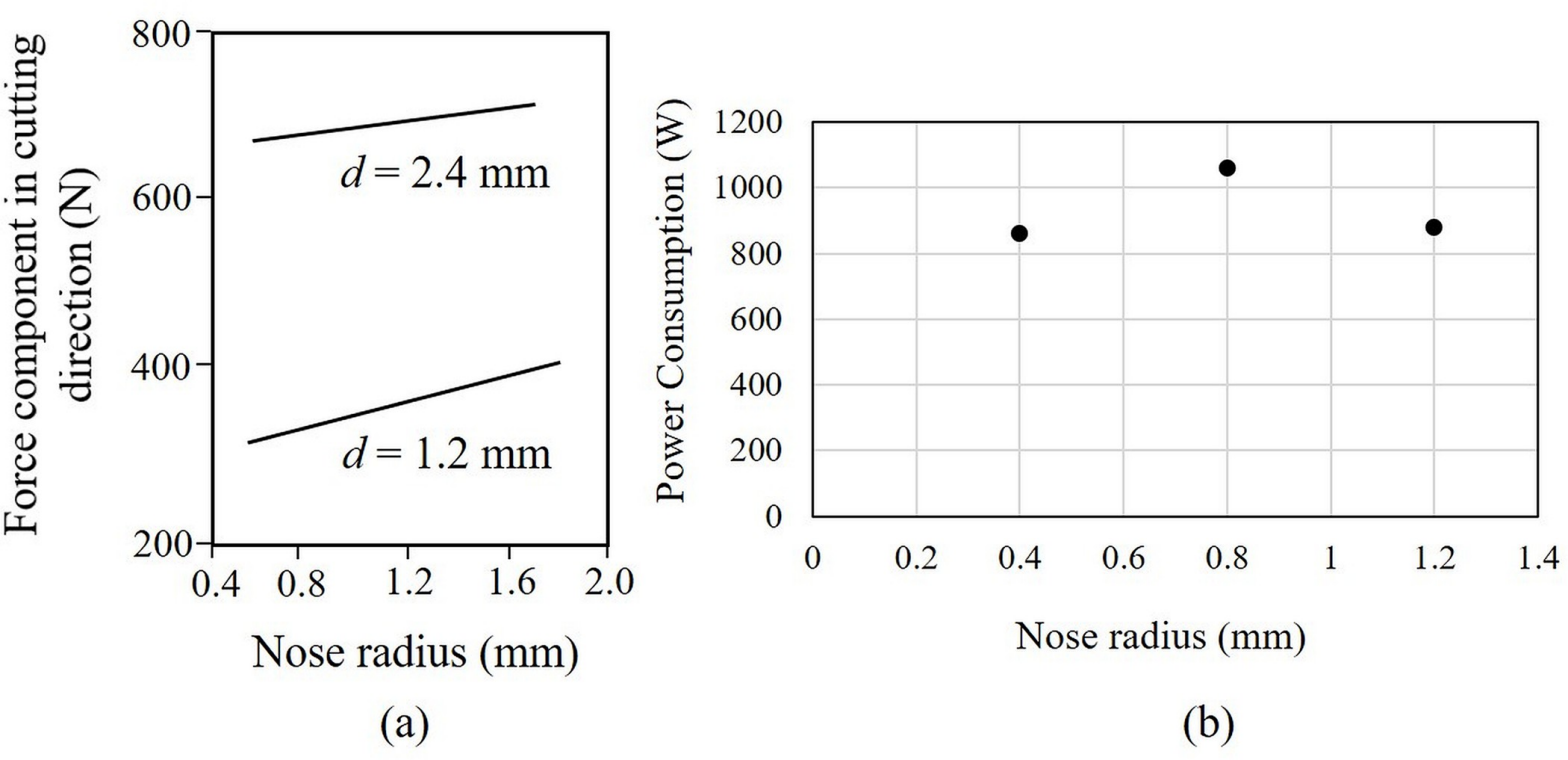
(a) Cutting force variation with nose radius (*d*-depth of cut) [34] and (b) effect of nose radius on power consumption during turning of AISI P-20 tool steel [35].

The presence of nose radius (*r*), one of the most important parameters in cutting tool geometry, affects the strength of the cutting edge and surface finish generated during cutting. A smaller nose radius will lower tool strength and increase the roughness of the machined surface in tandem with the usage of a worn tool [26]. On the other hand, a larger nose radius will generate chatter and vibration due to increased contact between tool and workpiece leading to increased cutting forces and power consumption, see Fig 5(A) and 5(B). Chatter is also aided by a cross-coupling effect between radial and axial vibrations during machining with cutters having larger nose radii [33]. This effect manifests as an unstable-stable chatter phenomenon in conjunction with process non-linearity, as reported by Rao and Shin [33].

An insert with a smaller nose radius of 0.1–0.2 mm is proposed for our cutter so as to reduce chatter vibration and cutting forces during turning. This is much smaller than the range of 0.8–1.5 mm recommended for conventional single-point tools based on the machining operation and workpiece material [27]. The reduced tool strength of the cutter due to lower nose radius is proposed to be compensated by a concomitant increase of thickness of the insert body coupled with a small *end cutting edge angle* (*C*_*e*_) and a moderate *side cutting edge angle* (*C*_*s*_). The possible loss in surface quality due to the application of a smaller nose radius will also be compensated for by the proposed values of *C*_*s*_ and *C*_*e*_.

### Surface texture

Surface texturing of tool rake face will be incorporated by producing micro or nano grooves using either *electric discharge machining* (EDM) or *laser beam machining* (LBM). These grooves will be filled with limited quantities of solid lubricant to ensure smooth machining sans the adverse effects of flooded lubrication, with its excesses, that is conventionally used. This technique is expected to bring down cutting forces, temperature and tool wear through reduced tool-chip contact length and enhanced lubrication that lower friction.

**Fig 6:**
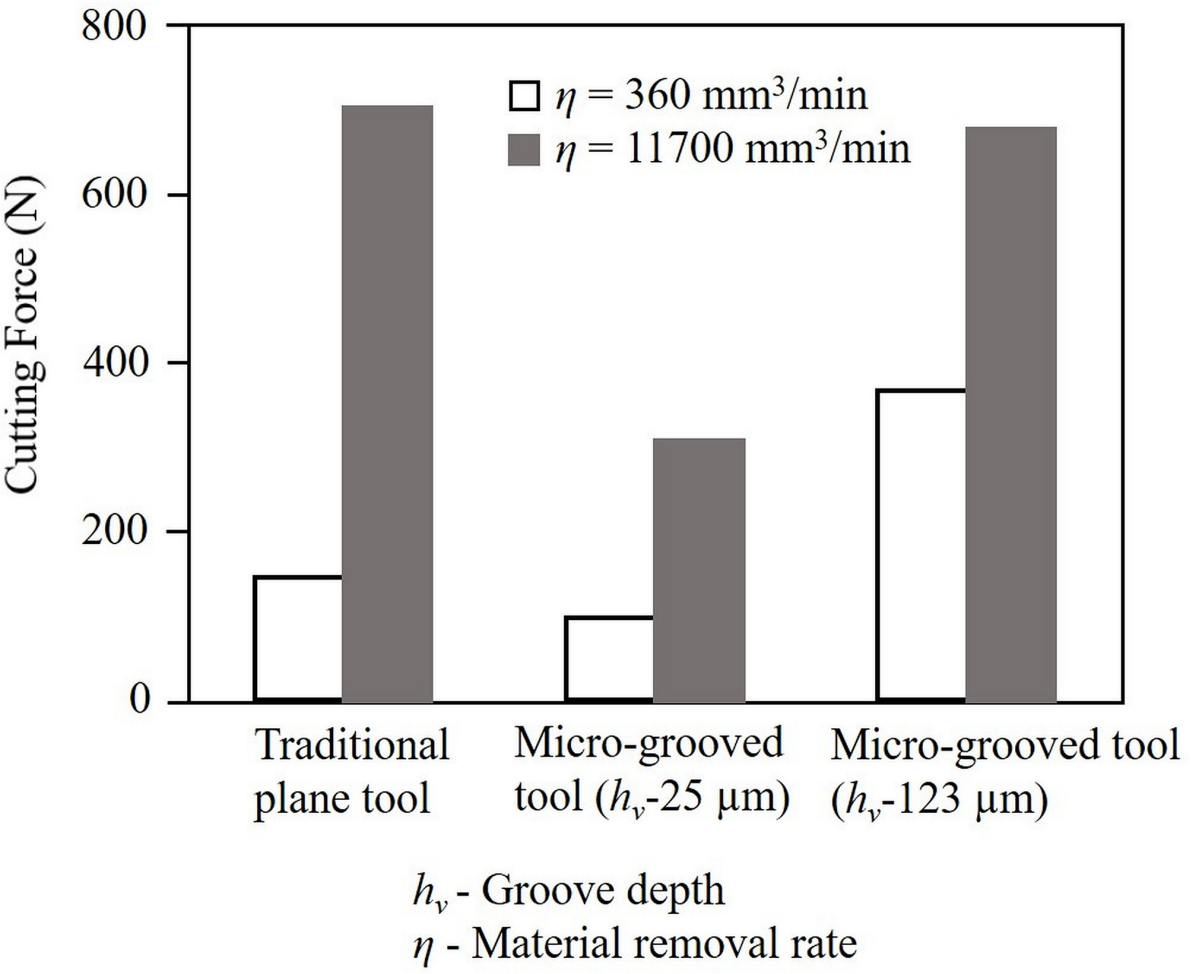
Cutting force variation with groove depth for plain and micro-grooved tool [17].

Xie et al. [17] reported a 32.7% reduction in cutting forces for a micro-grooved tool against a plain tool in the dry cutting of *titanium* (Ti-6Al-4V) alloy for two different material removal rates, see Fig 6. This force reduction was attributed to decreased tool-chip contact length due to texture that was filled with a low shear-strength solid lubricant that formed a film over the tool-chip contact zone [36–38]. The solid lubricant film was created due to the micro-grooves being filled with *molybdenum disulfide* (MoS_2_) solid lubricant. A decrease in cutting temperature was also reported during the machining of Ti-6Al-4V with micro-grooved tools [17]. The presence of grooves enabled heat dissipation through convection during the cutting process.

A caveat to note is that while reducing tool-chip contact length for limiting cutting forces and hence tool failure, surface texturing should ensure that the cutting-edge strength is not compromised. Rapid heat removal from the machining zone will ensure lesser wear for the textured tool. Additionally, the presence of solid lubricant in the micro-holes will reduce friction at the tool-chip interface by forming a lubricating layer in the contact zone. Even though surface roughness increases for a micro-grooved tool due to patterned grooves on the machined surface, the surface roughness deviation (defined as the maximum fluctuation of surface roughness from the mean value of five measured points) was seen to decrease [17]. It implied that a textured tool produced a more even surface as opposed to a traditional plain tool, even though the latter may have better surface quality. This result suggested a more stable cut with the micro-grooved tool producing less chatter and fulfilling the need for a chatter-reducing cutter design.

Xie et al. [39] reported a further 6.7% reduction in tool wear for grooves oriented along the direction of chip flow in contrast to grooves orthogonal to the chip flow direction. This reduction is reasonable since most of the heat generated in cutting operation is dissipated during chip formation. Optimum performance of a textured cutting tool was obtained for grooves parallel to the cutting edge in terms of reduced tool-chip contact length, reduced cutting forces, and lower friction [36, 40, 41].

Based on the above discussion, a cutting insert with triangular micro-grooves is proposed wherein the groove edges are parallel to the cutting edges of the insert, as shown in the 3D model of Fig 9(A). These grooves can be manufactured using a femtosecond laser machine. Obikawa et al. [40] reported that a texture pattern with smaller, deeper grooves and a reduced distance of the textured area from the cutting edge improved lubrication conditions and decreased cutting speeds. Following this, the geometrical dimensions of micro-groove and associated cutting parameters for optimum performance as obtained from the experimental investigations by Obikawa et al. [40] while machining Al6061-T6 alloy to compare the performance of textured against non-textured tools are listed below:

- Width of groove– 25 μm
- Depth of groove– 2 μm
- Distance of textured area from cutting edge– 100 μm

Additionally, the micro-grooves will be filled with a solid lubricant, i.e., MoS_2_, to investigate the collective effect of the texture dimensions and the solid lubricant.

### Mechanochemical effect

**Fig 7:**
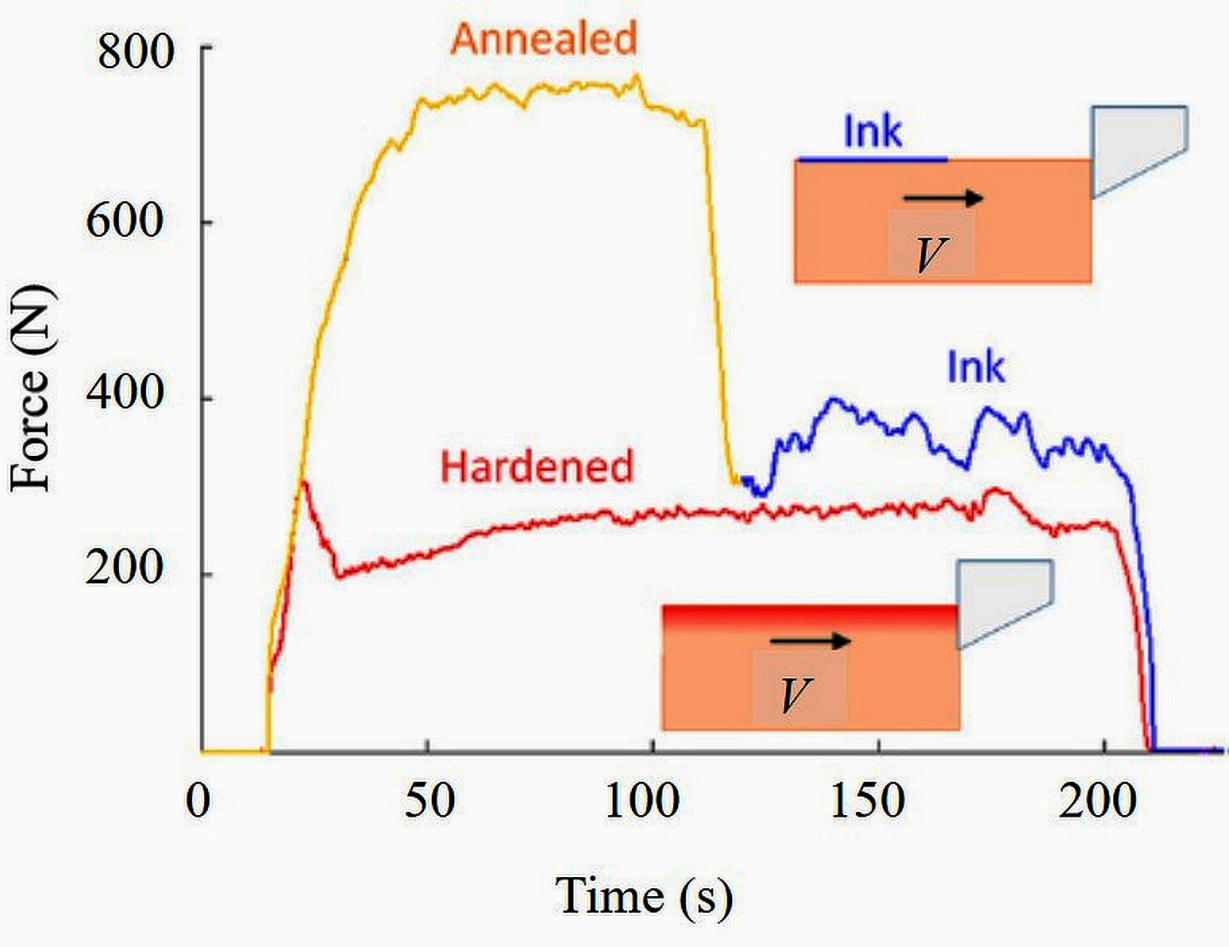
Comparison of cutting forces for annealed Cu (in yellow) with an ink coating half its length (in blue) and pre-strained Cu (in red) [20].

**Fig 8:**
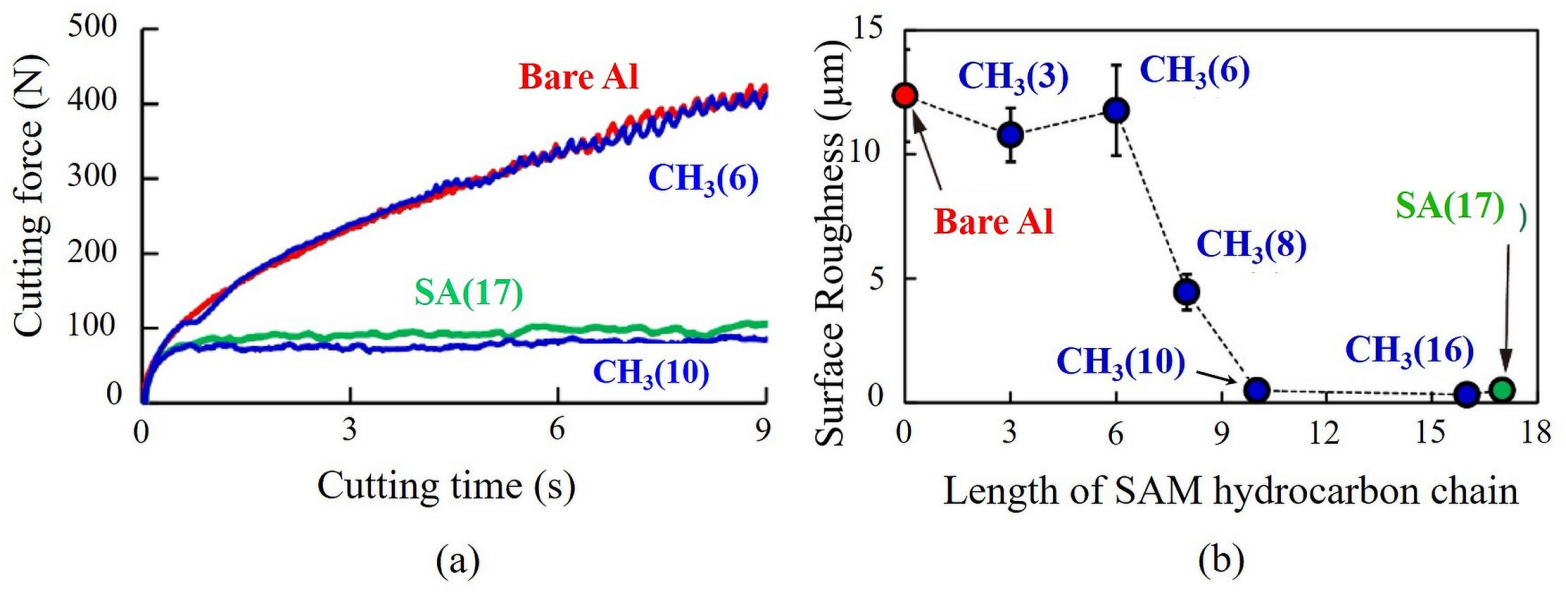
(a) Comparison of cutting force versus time for bare aluminum, Al and Al samples coated with self-assembled monolayer (SAM) films and (b) scatter plot for average surface roughness, *R*_*a*_ against length of SAM hydrocarbon chain [42].

Extensive efforts on the development, propagation and suppression of sinuous flow have been reported [20, 22–24]. In this regard, the *mechanochemical* (MC) effect seen as a result of adsorption of *surface active* (SA) media onto free surface has been demonstrated successfully for linear cutting of very ductile metals like *oxygen-free high conductivity copper* (OFHC Cu), *commercially pure aluminium* (Al 1100) and *iron* (Fe). Suppression of sinuous flow led to the reduction of large forces generated while cutting soft, annealed metals. Fold formation, a characteristic feature of sinuous flow, was suppressed by pre-straining the annealed work material, causing grain refinement, ductility-reduction and bringing about a 70% reduction in strain and hence cutting forces, as depicted in Fig 7. A similar suppression achieved by applying a *Dykem* ink layer over the metal surface just ahead of the cutting tool reduced cutting forces by more than 50%. The chemical media essentially brought about a local ductile-to-brittle transition coupled with significant cutting force reduction–a MC effect [22–24]. In other words, the MC effect of metal surface embrittlement induced by applying long-chain organic molecules arrested dislocation emission and thus, caused a transition from sinuous to segmented flow [42]. Consequently, this effect resulted in 85% reduction of cutting forces during plain-strain deformation of highly ductile Al 1100. Fig 8(A) illustrates this force reduction achieved by the application of long-chain *self-assembled monolayer* (SAM) films in contrast to shorter SAMs, which render cutting forces almost equal to cutting uncoated Al. The MC effect induced by these long-chain molecules also drastically improved surface quality. Fig 8(B) shows the variation of *average surface roughness* (*R*_*a*_) with SAM chain length. Upon successful application to real-time machining operations, the MC effect should bring about lower cutting forces and enhance the surface quality of machined products. Accordingly, the MC effect will be considered for incorporation into our cutter design.

## Model of cutter

**Fig 9:**
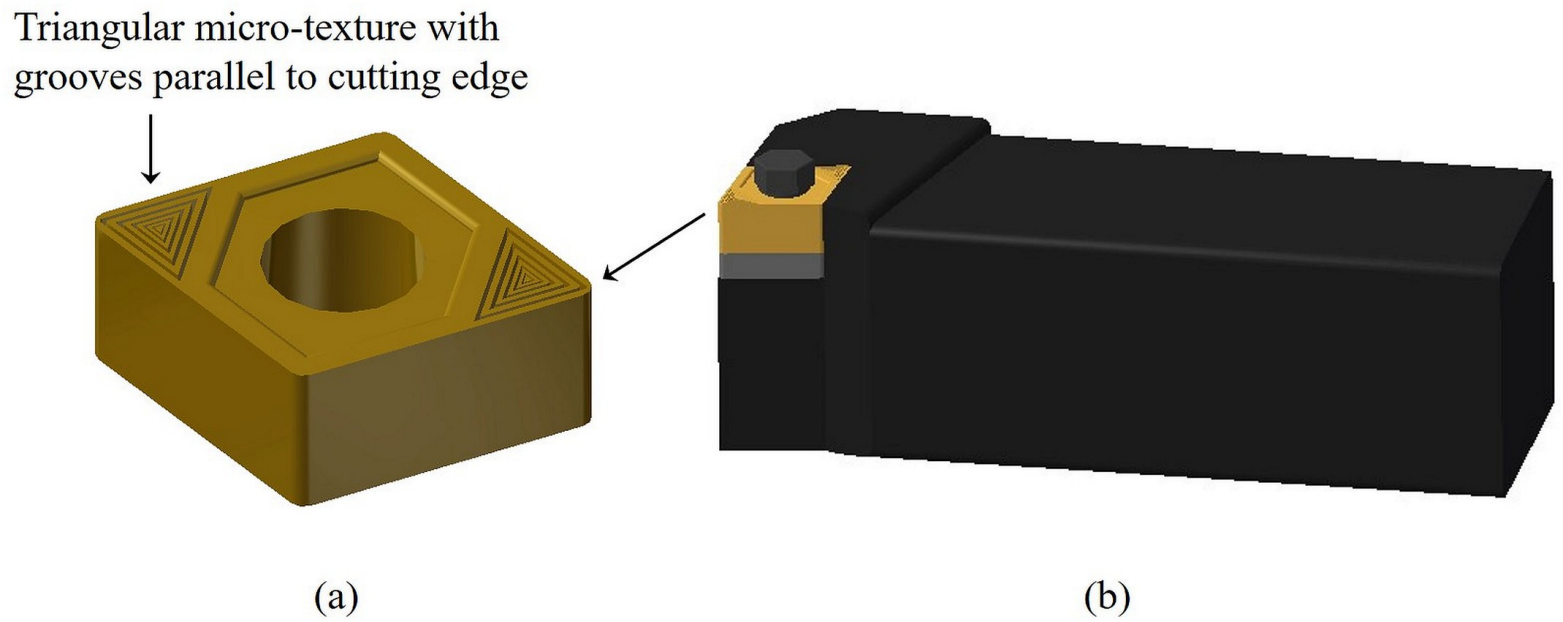
3D model of the (a) surface textured cutting insert with triangular micro-grooves and (b) proposed cutter generated using CATIA V5R19 software.

**Fig 10:**
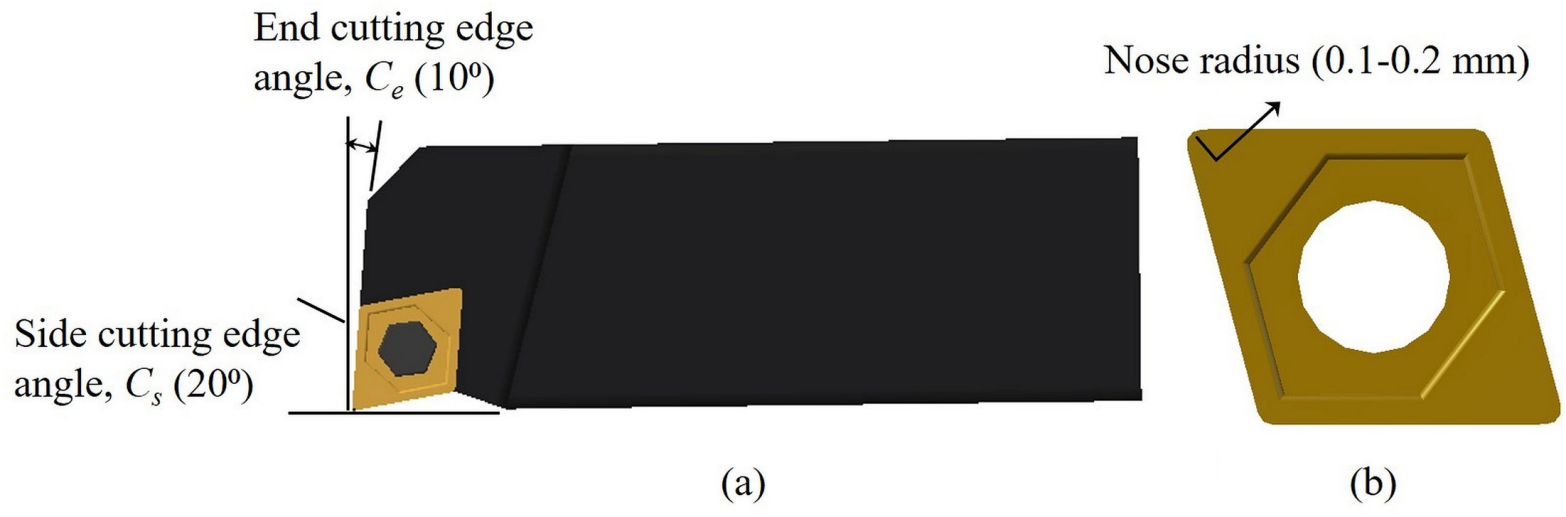
Top view of (a) turning cutter with proposed features of *C*_*s*_ and *C*_*e*_ and (b) insert with smaller nose radius.

The solid model for the proposed cutter design, shown in Fig 9(B), was created using CATIA V5R19 software. Fig 9(A) shows the proposed surface-textured insert with triangular grooves parallel to the cutting edges. In addition to these, the specific features of *side* and *end cutting edge angles* and smaller nose radius incorporated into our cutter design are also evident and clearly brought out in Fig 10(A) and 10(B). The insert is made of uncoated *tungsten carbide* (WC) and is clamped onto a *high-speed steel* (HSS) tool-holder possessing the proposed features. This cutter could be used to machine a range of materials, including aerospace alloys like Al6061, Ti-6Al-4V, and other metallic materials, when coupled with the *mechanochemical* effect.

## Experimental details

### Materials and experimental setup

Machining experiments were performed on a HAAS CNC lathe machine with cutting tools having variable *C*_*s*_ and *C*_*e*_ keeping all other tool geometry features like *α*, *γ* and *r* constant to study the effect of these angles on cutting forces. Table 1 shows the cutting parameters and cutting conditions under which machining was performed. These depicted values of *C*_*s*_ and *C*_*e*_ were selected from commercially available cutters by SECO tools so as to include their extremes and then study the force change therein.

**Table 1:** Cutting parameters used for the experiments.

Tool	*C*_*s*_ (°)	*C*_*e*_ (°)	*r* (mm)	Workpiece	Cutting speed (m/min)	Feed (mm/rev)	Depth of cut (mm)
SSDCN2525M09	45	45	0.4	Al-6061	10, 100	0.1, 1	0.1, 0.5, 1
SCLCR-16-3	-5	5					
SDHCR2525M11	-17.5	17.5					

In another set of experiments, commercially available cutting tools with variable *r* were used keeping *C*_*s*_, *C*_*e*_, *α* and *γ* constant to study their effect on cutting forces. Here too, inserts with a very small *r* of 0.2 mm and a higher *r* of 1.2 mm were selected along with an intermediate *r* of 0.4 mm for comparison encompassing the extreme values. For these tests, a constant *C*_*s*_ = -5° and *C*_*e*_ = 5° were selected.

**Fig 11:**
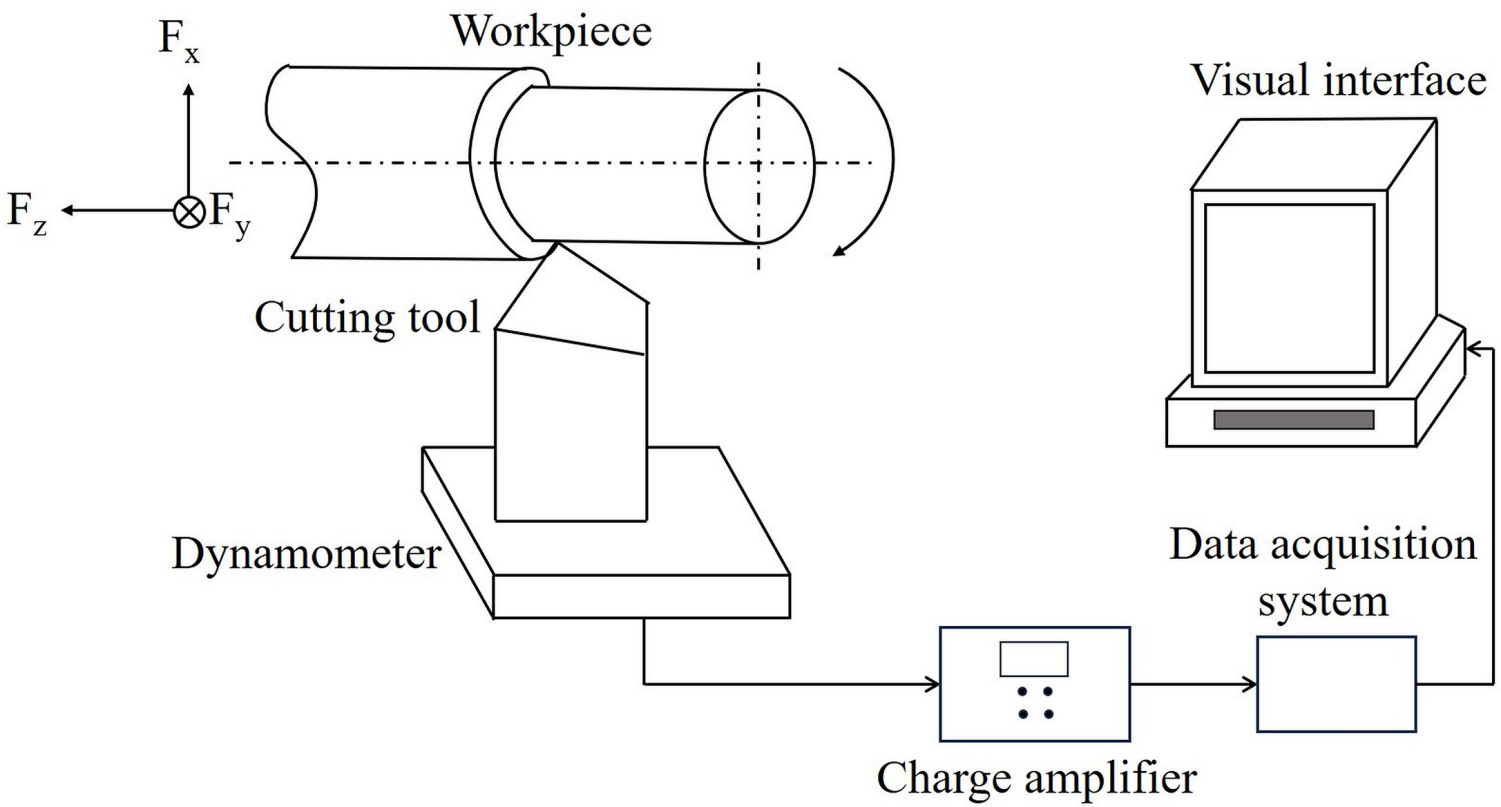
Machine-tool-workpiece-dynamometer experimental setup schematic.

The cutting conditions, depicted in Table 1, under which machining was performed were also selected to include a lower (*V* = 10 m/min) and higher speed (*V* = 100 m/min); low and very high feed, *f* of 0.1 and 1 mm/rev respectively; and depth of cut, *d* ranging between 0.1–1 mm. These are test conditions selected to study the effect of cutting edge angles and nose radius on cutting forces over a range beyond that recommended for the tool geometry used as per standards. Solid aluminum (Al6061) rods of 2-inch diameter were rigidly fixed onto the 3-jaw chuck of the lathe. The cutting forces were measured by mounting the tool holder on a three-axis Kistler 9257B dynamometer and feeding the cutting force signals into a Kistler 5010 charge amplifier connected to the dynamometer. These signals were recorded on a computer using data acquisition software after converting them to digital signals. Fig 11 shows the schematic of the machine-tool-workpiece-dynamometer setup.

### Statistical analyses

The experimental data obtained from the cutting tests described above were used for regression analysis to predict cutting forces using nose radius, side and end cutting edge angles as the independent variables. This was done since the proposed features are not commercially available and hence, experimental investigations for a combination of these particular values could not be performed. A polynomial regression model of degree 2 was identified as the best fit and this model was trained using the experimental dataset of measured cutting forces. Using MATLAB R2022b software, cutting forces for the proposed nose radius of 0.1 mm, side cutting edge angle of 20° and end cutting edge angle of 10° were predicted from this regression analysis.

Cutting forces vary with a range of conditions such as tool geometry features and cutting parameters like speed (*V*), feed (*f*) and depth of cut (*d*) to various degrees. In order to examine the effect of the tested tool geometry features along with cutting conditions on cutting forces, an analysis of variance (ANOVA) was performed. For this, the collected experimental data were arranged in a suitable format based on Taguchi’s L_16_ orthogonal array (OA). The objective was to determine the significance individually of the effects of nose radius, side and end cutting edge angles along with cutting conditions on measured forces. A multiway ANOVA was performed on MATLAB R2022b for two factors–one being the cutting condition and the other being the geometric parameter assessed–each factor having four levels. These process parameters and their corresponding levels are presented in Table 2. An alpha level of 0.05 was used to reject the null hypothesis. To ensure testing of all the levels of each of the parameters, an L_16_ orthogonal array was selected for maximum resolution [43].

**Table 2:** Process parameters and their corresponding levels.

Parameter	Symbol	Unit	Levels			
			Level 1	Level 2	Level 3	Level 4
Nose radius	*r*	mm	0.1	0.2	0.4	1.2
Cutting condition	*d*, *f*	mm, mm/rev	0.1, 1	0.5, 0.1	0.5, 1	1, 1
Side cutting edge angle	*C*_*s*_	°	-17.5	-5	20	45
Cutting condition	*d*, *f*	mm, mm/rev	0.1, 1	0.5, 0.1	0.5, 1	1, 1
End cutting edge angle	*C*_*e*_	°	5	10	17.5	45
Cutting condition	*d*, *f*	mm, mm/rev	0.1, 1	0.5, 0.1	0.5, 1	1, 1

## Experimental results

**Fig 12:**
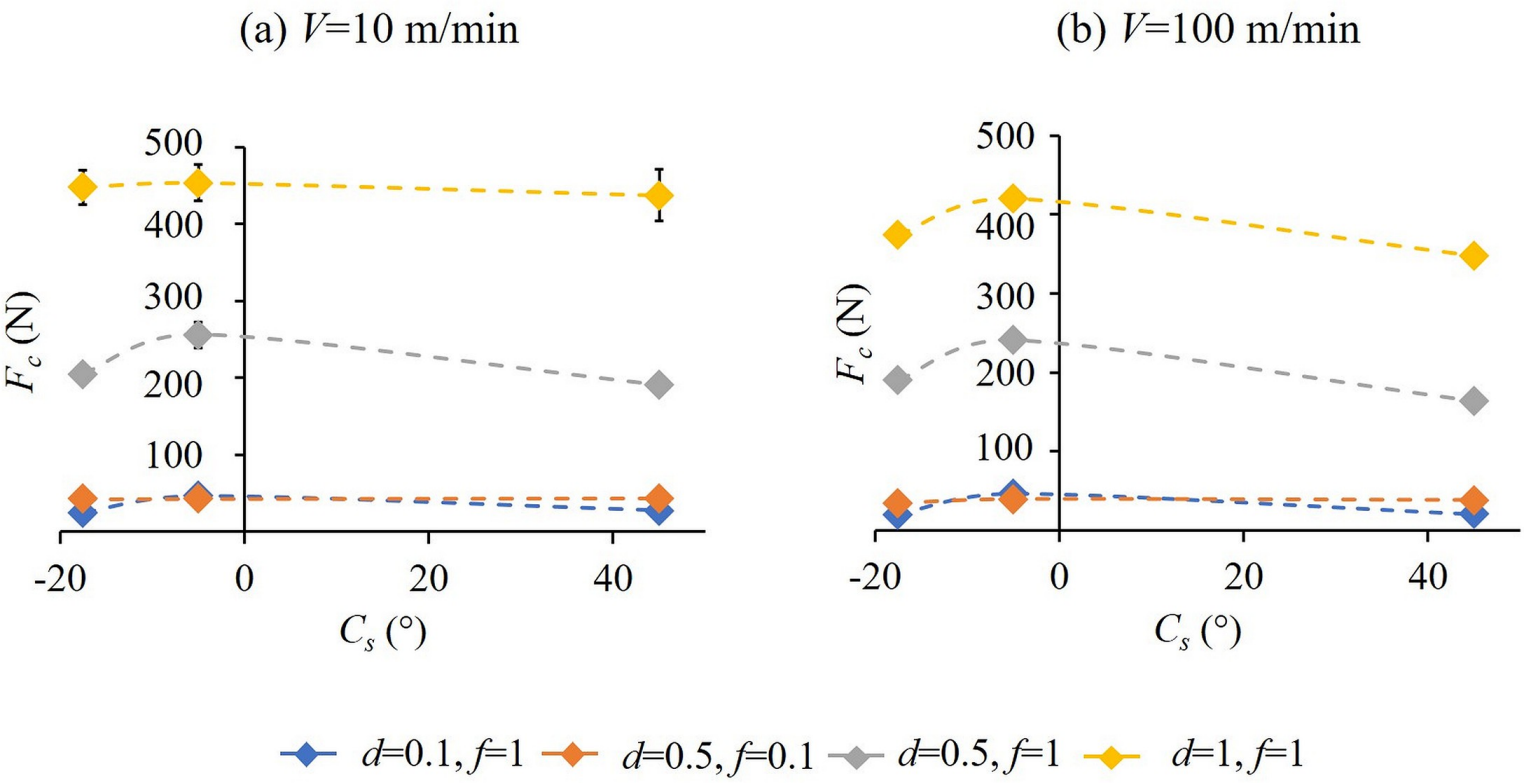
Variation of cutting force, *F*_*c*_ with side cutting edge angle, *C*_*s*_ at (a) *V* = 10 and (b) *V* = 100 m/min for different cutting conditions.

**Fig 13:**
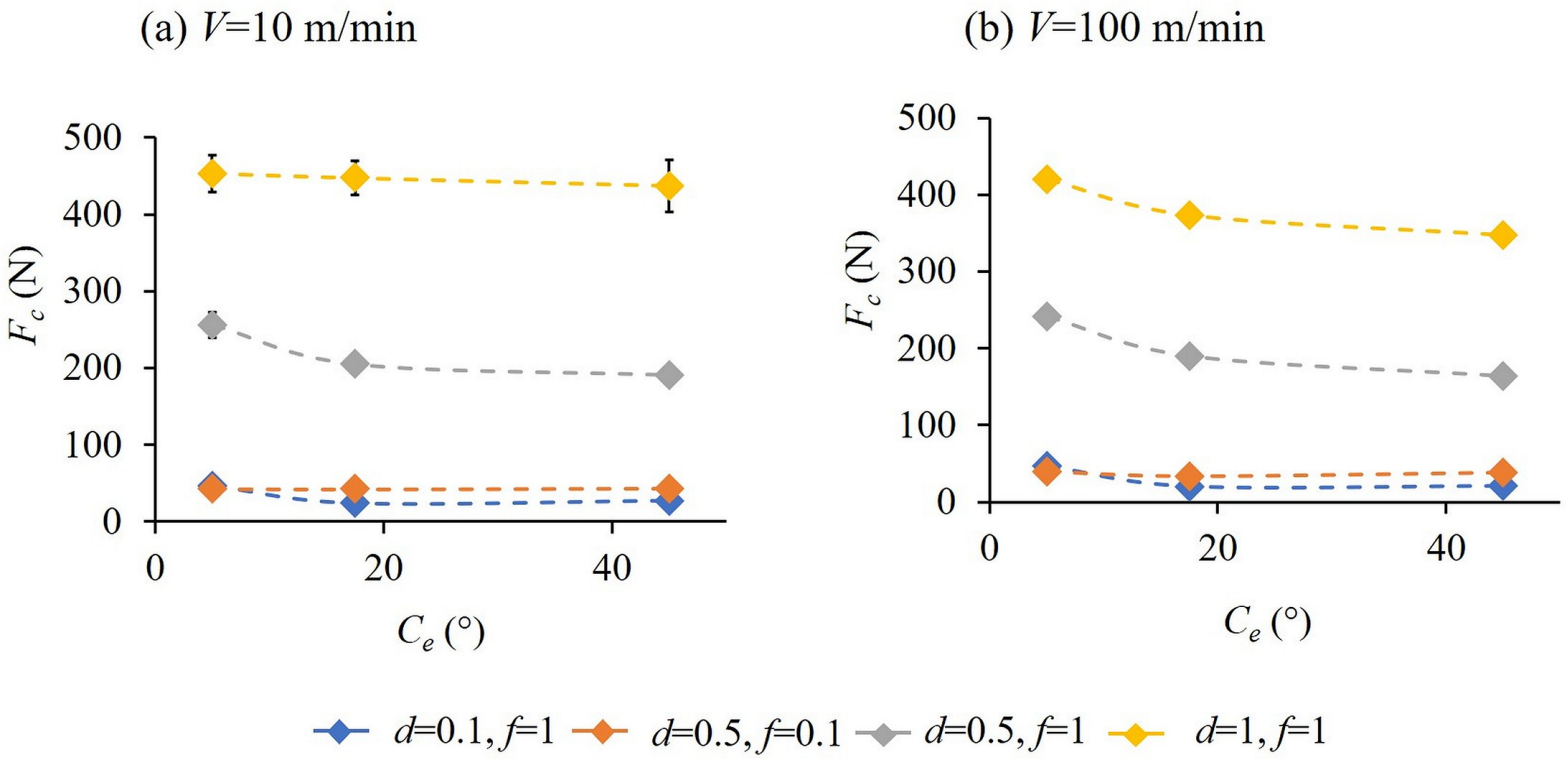
Variation of cutting force, *F*_*c*_ with end cutting edge angle, *C*_*e*_ at (a) V = 10 m/min and (b) V = 100 m/min for different cutting conditions.

Fig 12(A) and 12(B) illustrate the variation of cutting forces, *F*_*c*_ with *C*_*s*_ (-17.5°, -5° and 45°) at cutting speeds of 10 and 100 m/min respectively. With an increase in *C*_*s*_, *F*_*c*_ increases first and then starts to decrease. On the other hand, Fig 13(A) and 13(B) separately bring out the variation of cutting force, *F*_*c*_ with *C*_*e*_ (5°,17.5° and 45°) at the cutting speeds of 10 and 100 m/min respectively. Here, with an increase in *C*_*e*_ from 5°, *F*_*c*_ decreases and then starts to increase moderately. The minimum *F*_*c*_ for the lower feed and depth of cut combinations occurs at a *C*_*s*_ and *C*_*e*_ of -17.5° and 17.5° respectively. For cutting conditions including higher feed and depth, the minimum *F*_*c*_ was observed for the *C*_*s*_ = *C*_*e*_ = 45° tool. For almost all the cutting conditions the *C*_*s*_ = -5°, *C*_*e*_ = 5° cutting tool rendered relatively higher cutting forces. From the above, even though an increase in *C*_*s*_ or having a negative *C*_*s*_ might seem favorable for reduced cutting forces, there are limitations in terms of the direction of cutting in the former and loss of cutting edge strength due to decreased included angle between the cutting edges in the latter case, see Fig 2. Additionally, a smaller nose radius, also proposed as part of the tool design, increases the possibility of enhanced tool wear. In this case, having a fairly large included angle corresponding to non-negative *C*_*s*_ values will assist in maintaining the strength of the cutting tool edge.

**Fig 14:**
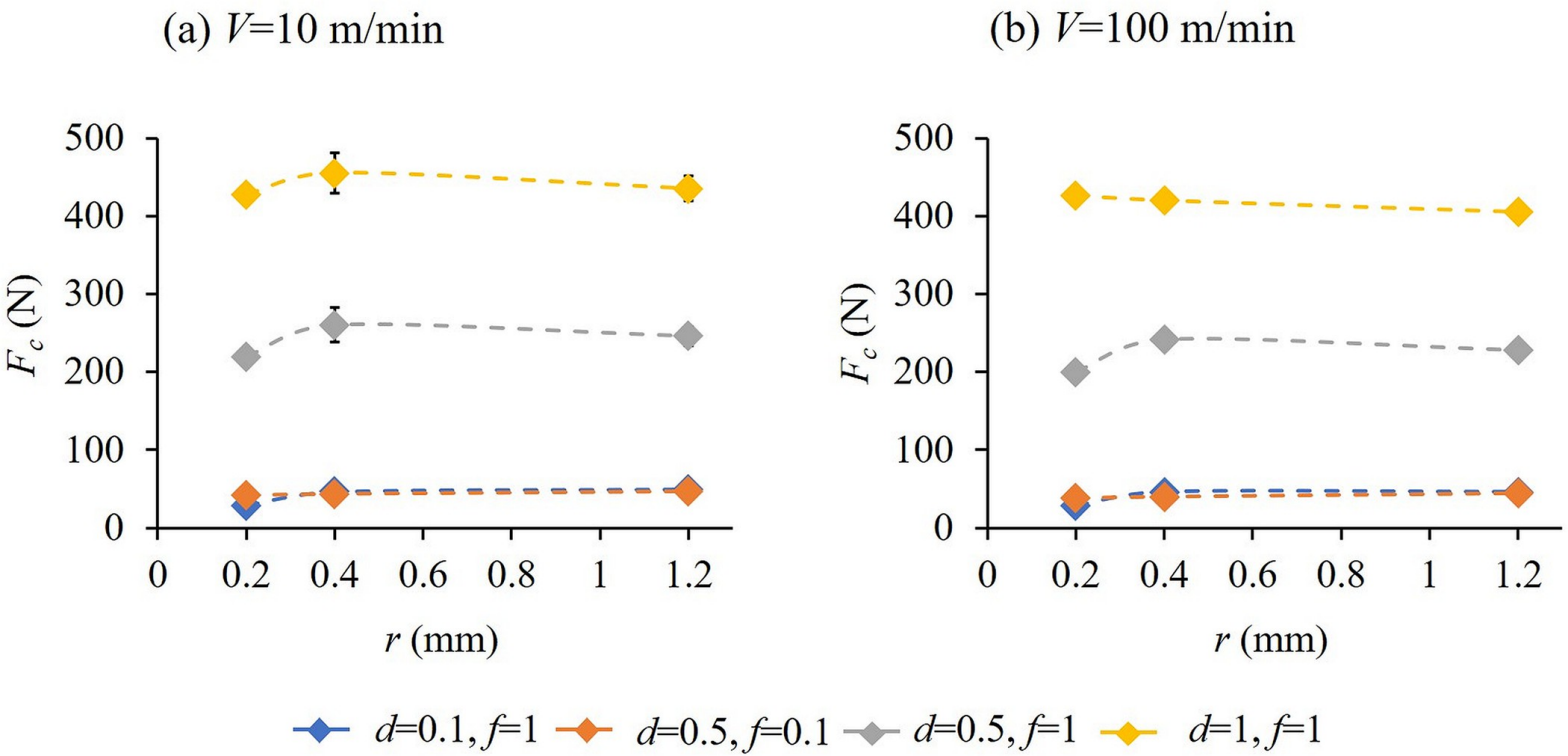
Variation of cutting force, *F*_*c*_ with nose radius, *r* at (a) *V* = 10 and (b) *V* = 100 m/min for different combinations of feed and depth of cut.

Cutting force, *F*_*c*_ is the least for the nose radius, *r* = 0.2 mm which is the smallest of the three nose radii tested, as seen in Fig 14(A) and 14(B) for cutting speeds of 10 and 100 m/min respectively for all cutting conditions except at *V* = 100 m/min, *f* = 1 mm/rev and *d* = 1 mm. In fact, this minimum force was observed at a feed much higher than that recommended for a nose radius of 0.2 mm [44]. This indicates that the proposed nose radii value of 0.1–0.2 mm will render lower cutting forces over a range of cutting conditions including more aggressive speeds and feeds and for machining material systems such as Ti- and Ni-based alloys with suitable tool materials.

## Statistical inference

Based on the experimental results for cutting force variation with nose radius and cutting edge angles presented above, regression analysis was performed to predict cutting forces for the proposed nose radius, side and end cutting angles for all the cutting conditions. A best-fit polynomial regression model of degree 2 was selected for the analysis. The regression model was trained using the nose radii and side and end cutting edge angles as the independent variables to predict the cutting force from the collected experimental data. The results from this regression analysis are presented in Table 3 for *r* = 0.1 mm, *C*_*s*_ = 20° and *C*_*e*_ = 10°. The predicted cutting force for the proposed nose radius of 0.1 mm shows as much as 65% decrease compared to the higher nose radius value of 1.2 mm (see Fig 14 and S1 Data) under certain cutting conditions. As opposed to this decrease for nose radius, the proposed side cutting edge angle of 20° rendered higher predicted cutting forces. However, this angle is present in conjunction with the end cutting edge angle on a cutter, as shown in Fig 2, and the proposed *C*_*e*_ of 10° generated reduced cutting forces. Hence, this *C*_*e*_ will compensate for the increased forces associated with *C*_*s*_ = 20°. A negative *C*_*s*_ entails decreased tool strength through reduced included angle between the cutting edges, see the tool geometry in Fig 2. On the other hand, a high positive *C*_*s*_ (eg. 45°) with its neutral orientation has limitations on the direction of cutting and machining operations. These caveats will be further amplified for use with a smaller nose radius, such as the one proposed, and hence, govern the selection of the proposed *C*_*s*_ of 20° that will maintain the tool strength without any limit on cutting direction or machining operation.

**Table 3:** Predicted cutting forces based on regression analysis for the proposed tool geometry values.

Cutting condition (*d*:mm; *f*:mm/rev) (d: mm, f: mm/rev) (d: mm, f: mm/rev) ()		*d* = 0.1, *f* = 1	*d* = 0.5, *f* = 0.1	*d* = 0.5, f = 1	*d* = 1, *f* = 1
**Cutting force (N) for the proposed values**	***V* = 10 m/min**				
	***r* = 0.1 mm**	17.4	40.2	192.95	408.4
	***C***_***s***_ **= 20°**	58.8	43.5	277.3	453.2
	***C***_***e***_ **= 10°**	35.8	43.1	232.3	451
	***V* = 100 m/min**				
	***r* = 0.1 mm**	17.5	36.4	172.65	429.7
	***C***_***s***_ **= 20°**	60	43.8	258.8	436.3
	***C***_***e***_ **= 10°**	34	36.7	217.8	399.4

In order to determine the significant parameters affecting the cutting forces, analysis of variance (ANOVA) was conducted based on Taguchi’s L_16_ orthogonal array. Two-way ANOVA was performed to study the effect of cutting conditions with each of the tool geometry parameters of nose radii I, side (*C*_*s*_) and end (*C*_*e*_) cutting edge angles. The four levels for the cutting conditions and tool geometry parameters of *r*, *C*_*s*_ and *C*_*e*_ are shown in Table 2. The experimental data was obtained according to Taguchi L_16_ orthogonal array to achieve the desired resolution based on two factors with four levels each. Table 4 shows the experimental cutting force results obtained for a combination of nose radii and cutting conditions at a cutting speed of 10 m/min.

**Table 4:** Experimental results for cutting force (At constant: *V* = 10 m/min, *α* = 0°, *C*_*s*_ = -5°, *C*_*e*_ = 5°).

Test No.	Parameters		Cutting force, *F*_*c*_ (N)
	*r*	*d*, *f*	
1	0.1	0.1, 1	17.5
2	0.2	0.5, 0.1	37.6
3	0.4	0.5, 1	241.3
4	1.2	1, 1	405.3
5	0.1	0.5, 0.1	36.4
6	0.2	0.5, 1	200
7	0.4	1, 1	420.2
8	1.2	0.1, 1	45.9
9	0.1	0.5, 1	172.65
10	0.2	1, 1	426.3
11	0.4	0.1, 1	45.9
12	1.2	0.5, 0.1	44.8
13	0.1	1, 1	429.7
14	0.2	0.1, 1	28.7
15	0.4	0.5, 0.1	39.7
16	1.2	0.5, 1	227.9

Following experimental investigations for a combination of *r* (and similarly, *C*_*s*_ and *C*_*e*_) and cutting conditions shown in Table 4, the results of ANOVA for cutting force are presented in Table 5 for *V* = 10 m/min. It can be seen from the *p*-values (Prob>F) that the side and end cutting edge angles do not have a statistically significant effect on cutting forces (*p*-value > 0.05). While cutting conditions have the most significant effect on cutting forces, this is followed by the nose radius which still has a statistically significant effect on the forces generated (*p*-value < 0.05). Concomitantly, the force reduction from a smaller nose radius is also more significant than any increase and decrease associated with the side and cutting edge angles respectively. However, these angles play a significant role in controlling the onset of chatter [26, 27, 32] and hence, are important to our cutter design, as detailed in the “Side cutting edge angle” and “End cutting edge angle” subsections above. ANOVA was also performed for a cutting speed of 100 m/min to test the effect of the tool geometry parameters and cutting conditions on cutting forces, see S2 Data. The results from this gave a *p*-value > 0.05 for the nose radius effect. On the other hand, *p*-value for each of side and end cutting edge angles was less than 0.05 implying a statistically significant effect from both these angles, while cutting conditions continued to have a significant effect for higher cutting speed. At this condition, the force reduction from *C*_*e*_ will compensate for any force increase for *C*_*s*_ since both angles demonstrate significance. The use of the proposed tool design features of nose radius and side and end cutting edge angles is thus, contingent upon the selection of suitable optimum machining parameters of speed, feed and depth of cut to achieve improved efficiency. Overall, the ANOVA analyses for both speeds highlight the significance of the geometric features of nose radius and cutting edge angles along with cutting conditions for designing a single-point cutter capable of suppressing parasitic mechanisms without any ancillary systems. In addition, the incorporation of the MC effect will further improve the efficacy of such a cutter.

**Table 5:** Analysis of variance (ANOVA) for cutting force (At constant: *V* = 10 m/min, *α* = 0°).

Source	Sum Sq.	DOF	Mean Sq.	F	*p*-value
**Nose radius effect**					
Nose radius	3191	3	1063.7	7.32	0.0087
Cutting condition	421075.9	3	140358.6	966.37	0
Error	1307.2	9	145.2		
Total	425574	15			
**Side cutting edge angle effect**					
Side cutting edge angle	3031.9	3	1010.6	3.04	0.0853
Cutting condition	450134.4	3	150044.8	451.45	0
Error	2991.2	9	332.4		
Total	456157.5	15			
**End cutting edge angle effect**					
End cutting edge angle	1492.8	3	497.6	3.04	0.0856
Cutting condition	452670.2	3	150890.1	920.34	0
Error	1475.6	9	164		
Total	455638.5	15			

## Discussion

The difference in cutting forces with the variation of cutting angles and nose radius may seem very less from the plots but in the field of metal cutting and manufacturing in general, even the smallest engineered reduction or variation of force in real-time machining could prove to be a significant step towards achieving sustainability. This is especially true for a design like the one presented in this paper where different cutter features are being consolidated along with the MC effect for bringing down chatter and cutting forces because finally their cumulative reduction will be considered. The cutter design features presented above have been proposed to improve the efficiency of turning processes in particular and, machining processes in general. Such an improvement in efficiency will entail reduced cutting forces and hence, lower energy consumption due to the alleviation of parasitic mechanisms. An extensive review of literature has revealed that parasitic mechanisms, such as chatter, tool wear and ploughing dissipate considerable energy and make the metal cutting process less energy-efficient. A systematic methodology to minimize the undesirable consequences of these mechanisms by possible suppression or minimization of chatter, surface texturing of cutting insert and suppressing sinuous flow is addressed in this paper. It should be noted that although parasitic mechanisms have been categorized, there can be an overlap between their mechanisms. For instance, increased tool wear could lead to increased chatter and cutting forces and thereby, continue to bring down the efficiency of machining processes. Minimizing these parasitic mechanisms and ideally avoiding them, affordably through a suitably designed cutter will unlock the significant potential of *frugal manufacturing* [25] for sustainable development.

A possible reduction of surface quality due to the tool’s sharp nose and surface texture and also diminished tool strength due to a smaller nose radius are some caveats for our proposed design. The tool strength reduction will be compensated by using a thicker insert body. The values for the *side* and *end cutting edge angles* have been carefully selected by considering chatter reduction through a controlled supply of vibratory energy and also clearance requirement between tool and work surfaces to account for any possible loss of surface integrity. Apart from these features of tool geometry, cutting conditions like speed, feed and depth of cut also play a significant role in determining the surface quality of the machined surface. The combination of high speed, low feed and a moderate depth of cut in cutting is considered favorable for a good surface finish [45–47]. However, it should be noted that power consumption consequently increases during high-speed machining. An optimum set of machining parameters for generating lower cutting forces and hence consuming lesser power and; producing better machined surface quality with a high level of machining accuracy will be identified. In fact, the use of suitable cutting conditions with our design framework will be the general theme that can be extended to other cutting operations such as milling.

Table 6 summarizes the force reduction reported for some of the features discussed above. As highlighted in this table, a maximum force-reduction of 85% due to MC effect-induced embrittlement was reported [42]. A micro-grooved tool demonstrated about 57% reduction in the resulting cutting force signature [17]. Just machining with a solid lubricant was seen to give a force reduction of 31% [48].

**Table 6:** Energy savings, reported in literature, from schemes controlling parasitic mechanisms in metal cutting.

Sl. No.	Method		Tool	Workpiece	Cutting conditions (*V*:m/min; *f*:mm/ rev)	Force (N)	% reduction
1	Micro-grooving on rake face [17]	Plain tool	WC-Co	Ti-6Al-4V	*α*:0⁰, *V*:36, *f*:0.1	700	-
		Micro-grooved tool				300	57.1
2	Machining with solid lubricant [48]	Flood cooling	WC	Steel	*α*:-6⁰, *V*:110, *f*:0.25	650	-
		Solid lubricant (Graphite, Boric acid)				450	30.7
3	Mechano-chemical effect [42]	Bare Al	WC-Co	Al 1100	*α*:10⁰, *V*:0.3	600	-
		Long-chain SAM coated Al				90	85

**Fig 15:**
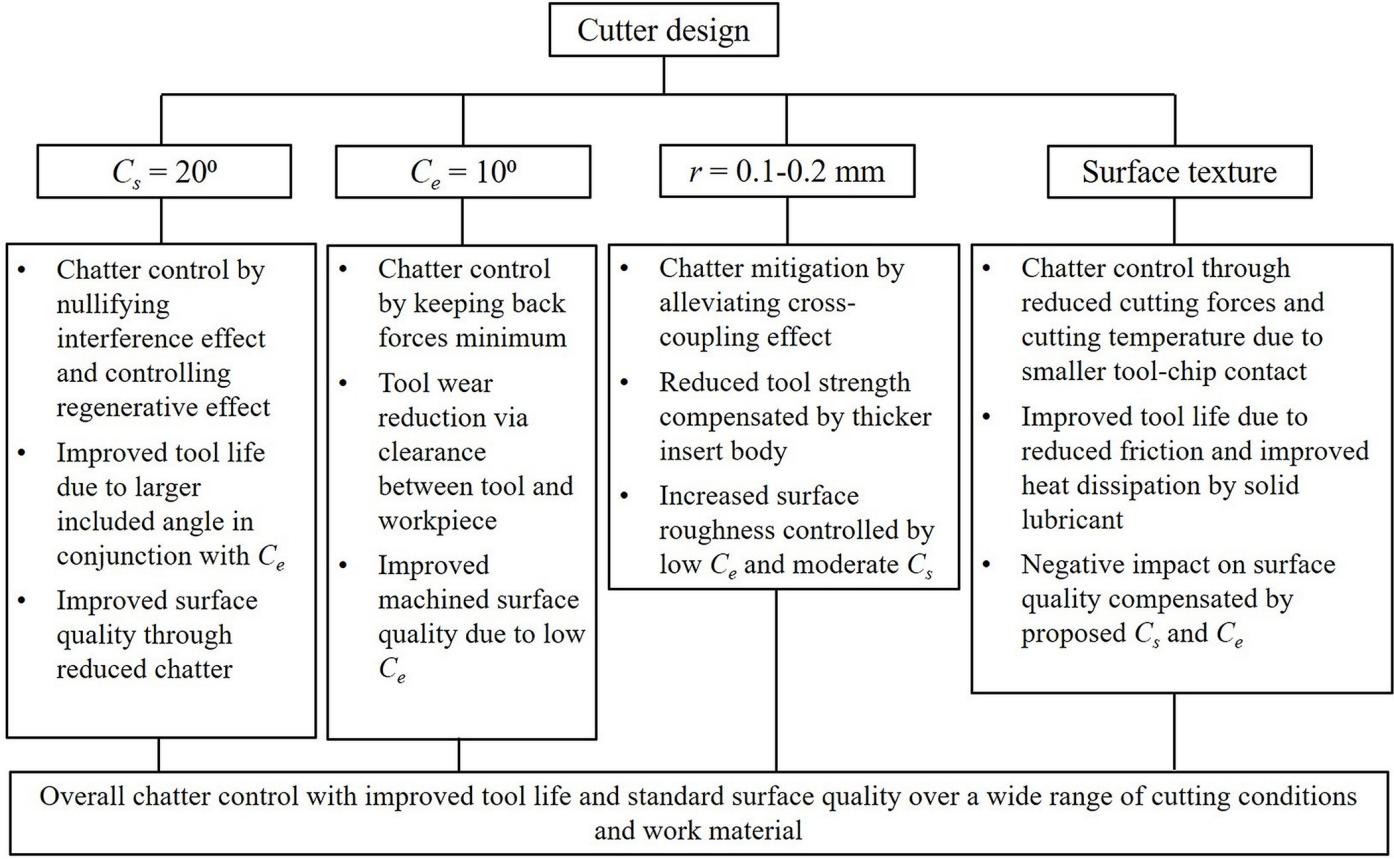
Flow chart depicting collective performance of proposed cutter design in combating parasitic mechanisms [23, 27, 32, 33].

Fig 15 depicts a flow chart representing the expected collective performance of the proposed cutter design features in controlling parasitic mechanisms. The *side* and *end cutting edge angle* values by themselves are expected to mitigate chatter, reduce cutting forces, tool wear and improve surface finish. A sharp cutting insert with a small nose radius contributes towards chatter control by alleviating the cross-coupling effect between radial and axial vibrations. Additionally, the incorporation of texture or micro-grooves onto the rake face of the cutter and filling these grooves with solid lubricant will also bring about reduced cutting forces and through that, reduce chatter. Ergo, a combination of these features of *side* and *end cutting edge angles*, smaller nose radius and surface texturing of cutting insert will mitigate overall chatter by contributing individually to chatter control. Both these *cutting edge angles* will also improve tool life and reduce tool wear by providing the necessary clearance between the contacting surfaces and forming a larger included angle between the cutting edges which is a critical factor considering the small nose radius proposed. Surface texturing too will improve tool life through reduced friction and improved heat dissipation from the tool-chip contact zone. However, with the reduction in nose radius, the tool strength and thereby, tool life might diminish. This loss in tool strength will be compensated by using a thicker insert. The combination of the proposed *side* and *end cutting edge angle* values with surface texturing on the rake face of the insert could, thus, bring about reduced tool wear. Furthermore, any possible loss of tool life due to the sharp nose of the insert is also considered for alleviation through the use of a thicker insert body. As illustrated in Fig 15, the proposed *C*_*s*_ and *C*_*e*_ values will individually improve surface finish through reduced chatter and vibration and enhanced clearance between the tool and work surfaces. This is another instance where the alleviation of the parasitic mechanism of chatter inherently takes care of the loss of surface integrity. However, a smaller nose radius and surface texturing are detrimental to favorable surface integrity of the machined surface. But again, the improvement in surface quality obtained from the proposed *C*_*s*_ and *C*_*e*_ could compensate for this loss arising from the nose radius and texture features. Thus, a combination of all these features is expected to render a good machined surface quality. In addition to the features depicted in Fig 15, the *mechanochemical* effect, being an independent mechanism, could alter the mechanics of our cutter design. This effect, shown to drastically reduce cutting forces while improving the surface finish, is expected to work favourably with the cutter design features in controlling the parasitic mechanisms.

The amalgamation of the features mentioned above has not been reported to date. Through the cutter design, the authors want to address the drawbacks caused by parasitic mechanisms in metal cutting. Accordingly, this work has united various geometric features with the MC effect through the proposed cutter design as a solution to these issues related to parasitic mechanisms. Of course, the cutter design itself is not full-proof and the authors do not claim that it can be used for all kinds of work material or all possible cutting conditions to achieve the same result. The various combinations of the features in the proposed cutter design including the MC effect are presented as a solution to bring down cutting forces and chatter by some margin individually and hopefully even more collectively. Detailed experiments are being undertaken to further study the collective effect of the features upon which the tool itself will be fabricated for final measurements.

## Conclusion

A novel turning cutter design is presented, based on the physics of *metal cutting*, for reducing cutting forces and hence energy consumption. Parasitic mechanisms such as chatter and tool wear leading to loss of surface integrity accompanied by increased cutting forces and specific cutting energy have hitherto eluded researchers and practitioners from achieving an efficient manufacturing process. The limitations from such parasitic mechanisms have necessitated a simple yet effective cutter design to minimize (or ideally suppress) them. Accordingly, our cutter design proposes features such as optimum *side* and *end cutting edge angles*; smaller nose radius accompanying a thicker insert body and; surface texturing on rake face, all of which are expected to collectively contribute towards minimization of power consumption.

The geometric features of nose radius (*r*), *side* (*C*_*s*_) and *end (C*_*e*_*) cutting edge angle*s were tested for the cutting of Al6061-T6 at a range of cutting conditions of speed, feed and depth of cut. Based on the data collected from these experimental investigations, regression analysis was performed to predict cutting forces for the proposed *r* of 0.1 mm, *C*_*s*_ of 20° and *C*_*e*_ of 10°. The predicted results revealed that the proposed nose radius and end cutting edge angle gave reduced forces whereas the side cutting edge angle led to increased forces. Analysis of variance (ANOVA) was performed to determine the significance of the effect of these geometric parameters along with cutting conditions on cutting forces. At the lower speed, the cutting edge angles did not have a significant effect and hence, the force reduction from the proposed nose radius overcomes any force increase due to the *C*_*s*_ of 20°. On the other hand, at the higher speed, both the cutting edge angles had a statistically significant effect on cutting force but the force reduction from *C*_*e*_ attenuates the force increase due to *C*_*s*_ here. Hence, there is an overall force reduction from the proposed geometric features at all the cutting conditions tested. The authors explain why the *C*_*s*_ value of 20° is still critical to maintain the cutting tool strength as well as machined surface integrity considering the smaller nose radius of the cutter. In addition, surface texturing of the cutting insert and incorporation of the *mechanochemical* effect will further bring down cutting forces, temperatures and strains and improve surface quality. From the ANOVA results that establish the significance of all the parameters at different cutting conditions, it is expected that the features proposed in the new cutter design will collectively bring about a significant amelioration of cutting forces and hence, energy consumed. Through this, the proposed cutter will contribute to sustainable and *frugal* manufacturing to alleviate the impact of *climate change*. The use of a single cutter avoids/minimizes deployment of ancillary systems to ward off parasitic mechanisms. In doing so, a single cutter reduces costs while maintaining quality thereby giving a *frugal* solution to sophisticated applications involving metal cutting. Application of the cutter to the machining of conventional metals and their alloys will open up scope for application in aerospace, structural, energy, biomedical and defense domains.

## Supporting information

S1 Data Measured cutting forces for nose radii, side and end cutting edge angles at all cutting conditions tested. (XLSX)

S2 Data Analysis of variance (ANOVA) for cutting force at constant *V* = 100 m/min, *α* = 0°. (XLSX)

S1 File (PDF)
