# Supplementary material for: A cutting tool architecture designed to address the parasitic mechanisms consuming excess power during machining and manufacturing operations–A review-based study towards sustainable manufacturing
Source: PLoS One. 2024 Apr 16;19(4):e0300132. doi: 10.1371/journal.pone.0300132 (PMC11020480; doi:10.1371/journal.pone.0300132)
Supplement: S1 File — (PDF) [file pone.0300132.s003.pdf]

SUPPORTING INFORMATION

Links to data for this paper:

https://zenodo.org/doi/10.5281/zenodo.10439504
https://zenodo.org/doi/10.5281/zenodo.10437570
https://zenodo.org/doi/10.5281/zenodo.10437637
https://zenodo.org/doi/10.5281/zenodo.10437629
